# A patient with macrodystrophia lipomatosa bilaterally affecting the entire upper extremity: reporting of a rare case and literature review

**DOI:** 10.1080/23320885.2021.1872380

**Published:** 2021-06-04

**Authors:** Kyoko Baba, Shinya Kashiwagi, Mitsuru Nemoto, Akira Takeda, Keizo Fukumoto, Eiju Uchinuma

**Affiliations:** a Department of Plastic Surgery, Kitasato University Medical Center, Saitama, Japan; b Department of Plastic and Aesthetic Surgery, School of Medicine, Kitasato University, Kanagawa, Japan; c Saitama Hand and Microsurgery Institute, Saitama, Japan

**Keywords:** Macrodystrophia lipomatosa, bilateral, entire upper extremity, median nerve

## Abstract

The patient, a 58-year-old Asian female, had the progressive, bilateral overgrowth of the entire upper extremity since her childhood and has undergone debulking surgery twice in her country. However, overgrowth progressed after surgery. The patient was diagnosed with Macrodystrophia lipomatosa (MDL) by physical and imaging findings in our departments.

## Introduction

Macrodystrophia lipomatosa (MDL) is a rare, nonhereditary, and congenital disorder that was first reported by Feriz [1] in 1925. The etiology and pathology of MDL are still not elucidated because of its rarity. MDL is characterized by the localized overgrowth of the extremities, with the disproportional, progressive proliferation of all mesenchymal elements [2]. In the vast majority of patients, overgrowth is unilateral and localized in the fingers or toes [2–4]. We encountered a patient with MDL bilaterally affecting the entire upper extremity who has never been reported in a medical journal, to the best of our knowledge. Here, we report on our patient with MDL bilaterally affecting the entire upper extremity, along with literature review.

## Case report

A 58-year-old Asian female, 149 cm in height and 79 kg in weight.

### Chief complaint

Bilateral overgrowth of the upper extremities.

### History of present illness

The patient has shown the bilateral overgrowth of the upper extremities since her childhood and has underwent debulking surgery (details unknown) at a hospital in her homeland to reduce tissues twice before coming to Japan. However, overgrowth progressed after every surgery, and pain caused by arm weights persisted. At age 58, the patient visited Japan to obtain the diagnosis and was examined at our departments.

### Family history

To the extent of her knowledge, none of her relatives had a congenital anomaly of the extremities.

### Clinical course

The patient did not desire any invasive tests and underwent whole-body computed tomography (CT) and magnetic resonance imaging (MRI) of the upper extremities.

The patient was diagnosed with MDL based on imaging results and returned to her homeland because follow-up was recommended. The patient did not desire anymore surgery, because of twice recurrences after debulking surgeries.

### Findings

#### Physical findings.

**Figures 1.**
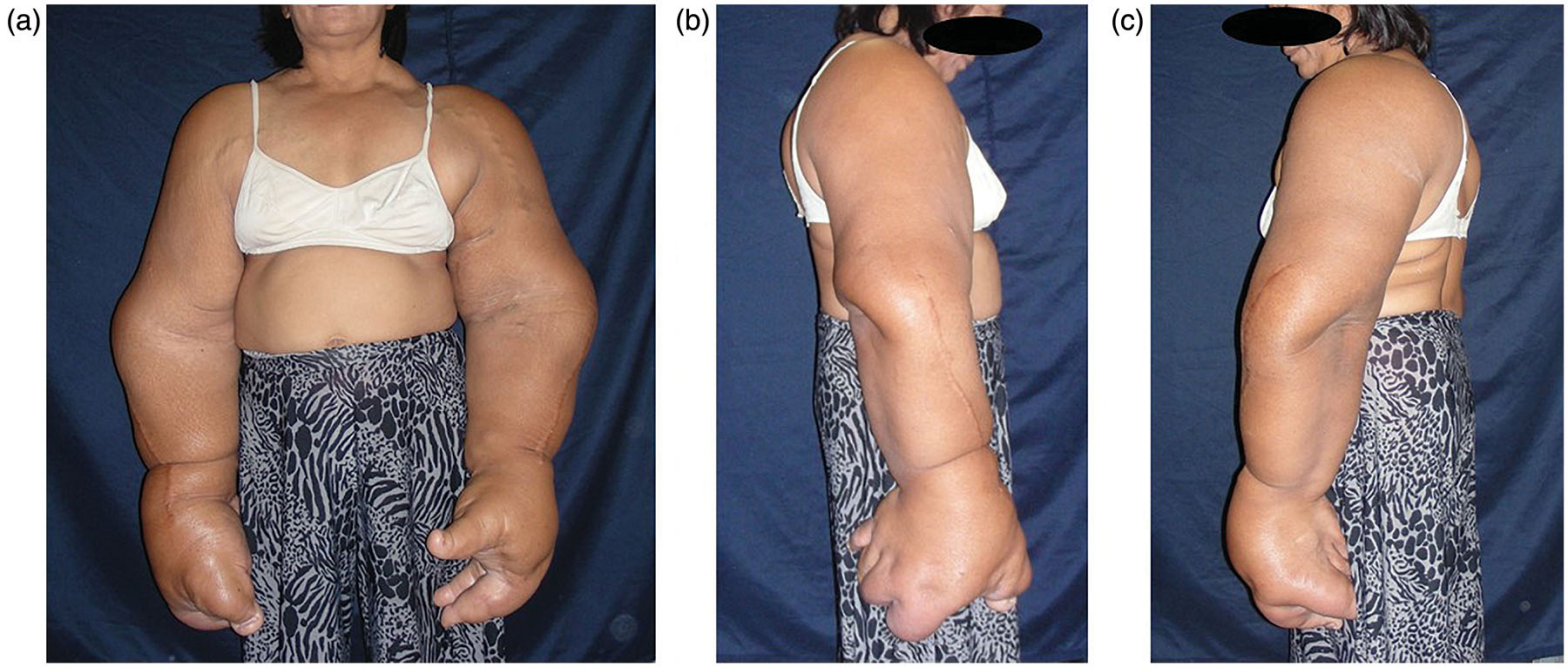
(a–c) Physical findings: The bilateral overgrowth of the upper extremities occurred, extending from the shoulders to the fingers. Both the length and circumference of the upper extremities increased.

**Figures 2.**
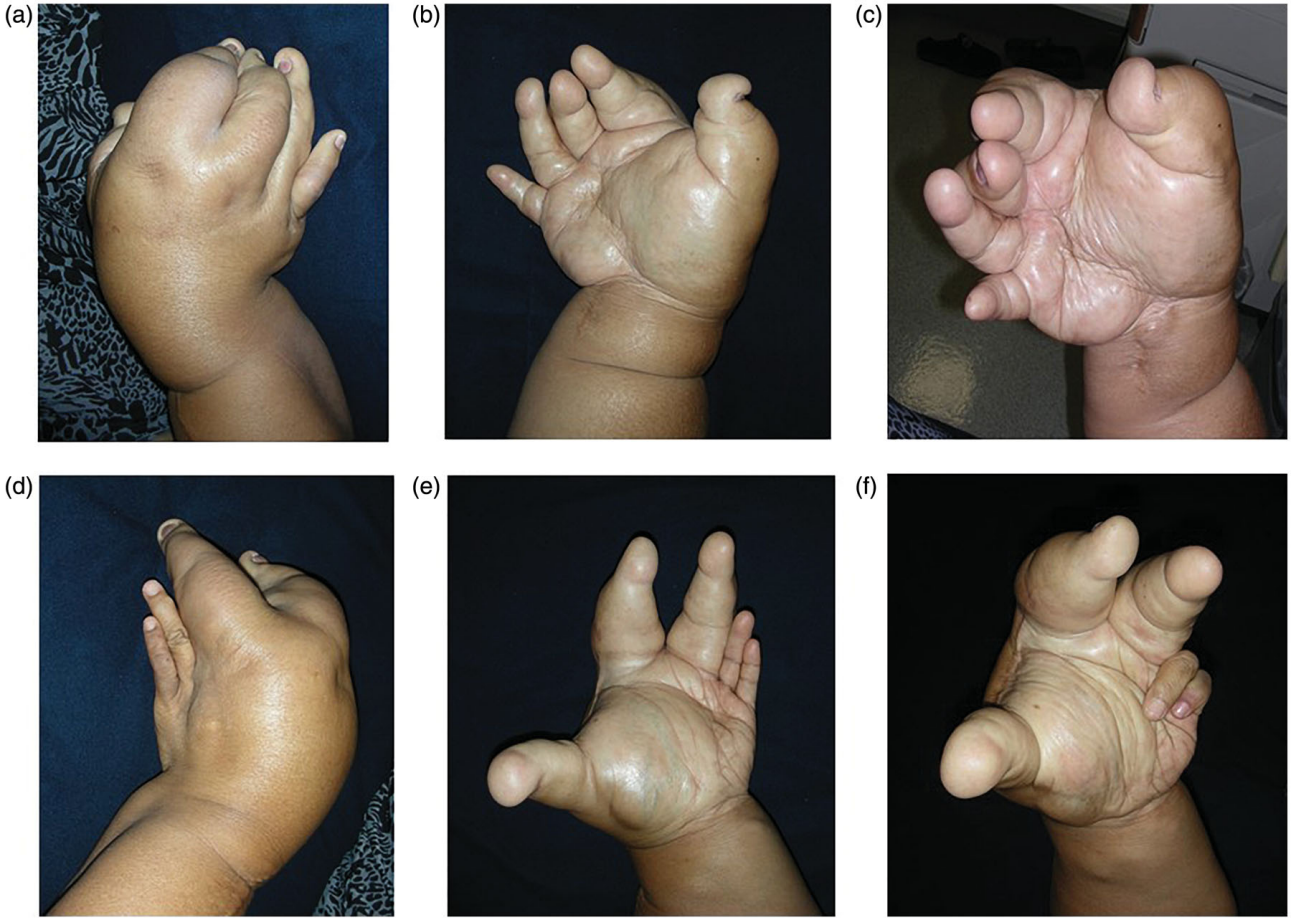
(a–f) Physical findings: The overgrowth of the ring and little fingers, as well as of the palm corresponding to the fourth and fifth metacarpal bones was not obvious in the left hand.

The bilateral overgrowth of the upper extremities occurred, extending from the shoulders to the fingers (Figures 1 and 2). Both the length and circumference of the upper extremities increased. The overgrowth of the ring and little fingers, as well as of the palm corresponding to the fourth and fifth metacarpal bones was not obvious in the left hand. Any congenital anomalies (e.g. syndactyly) were not found, except the bilateral, disproportional overgrowth of the upper extremities. Surgical cicatrices were found in the extensor aspect of the radius of both upper extremities, in an area between the flexor aspect of the right wrist joint and the palm of the right hand, and in the first interdigit of the left hand.

#### Physical examination

We could not measure range of motion on severe overgrowth-limb. We checked two-point discrimination (2PD) of her fingers, but it was not strict because of deformity. We could got only one 2DP each on five finger-tips, as below; Right Thumb 6 mm, Right index-finger 9 mm, Right ring-finger 13 mm, Left ring-finger 4 mm, Left little-finger 5 mm.

#### Imaging findings

##### CT scans

**Figures 3.**
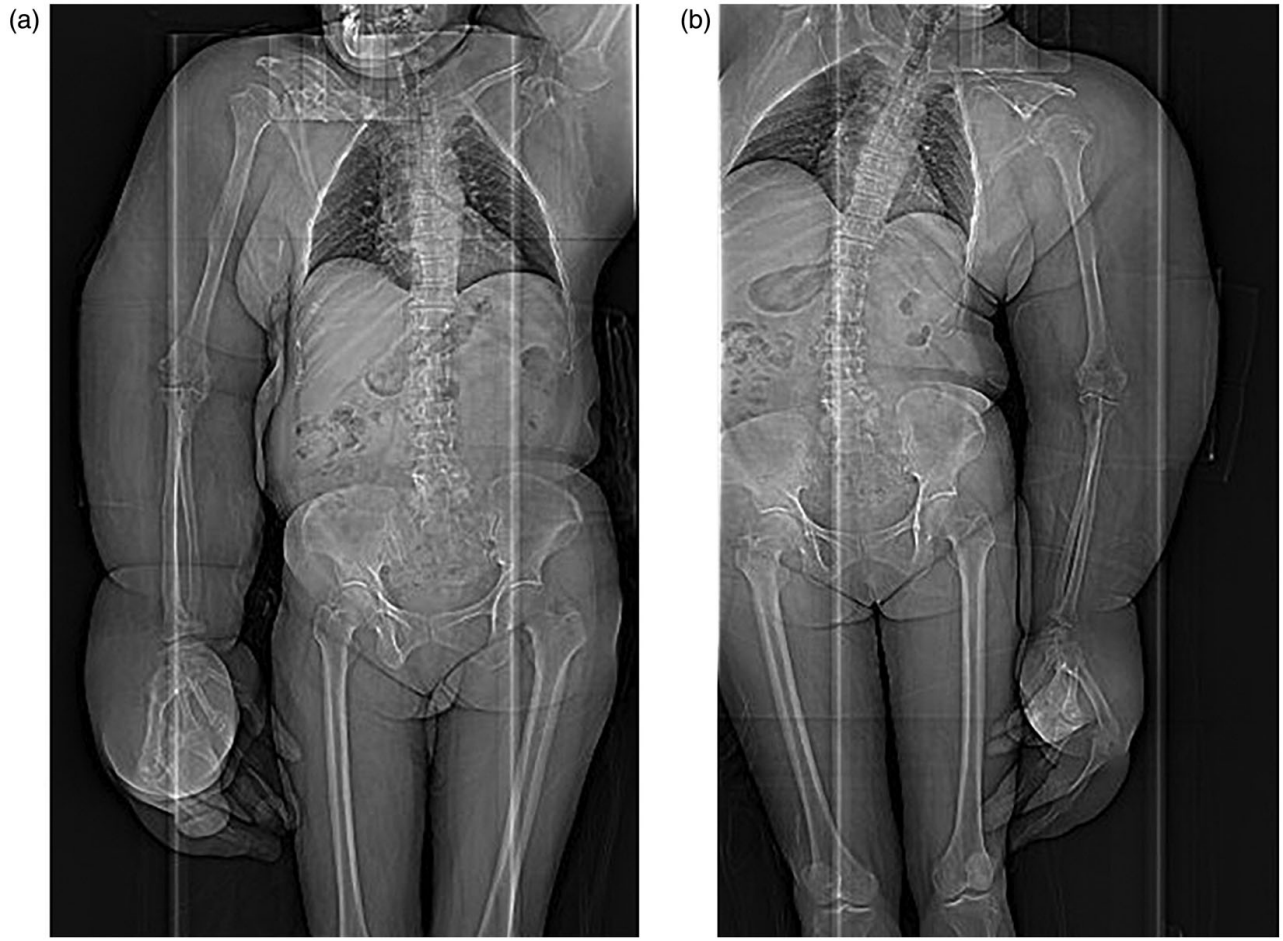
(a,b) CT scans: Any congenital anomalies (e.g., syndactyly) were not found, except the bilateral, disproportional overgrowth of the upper extremities.

**Figures 4.**
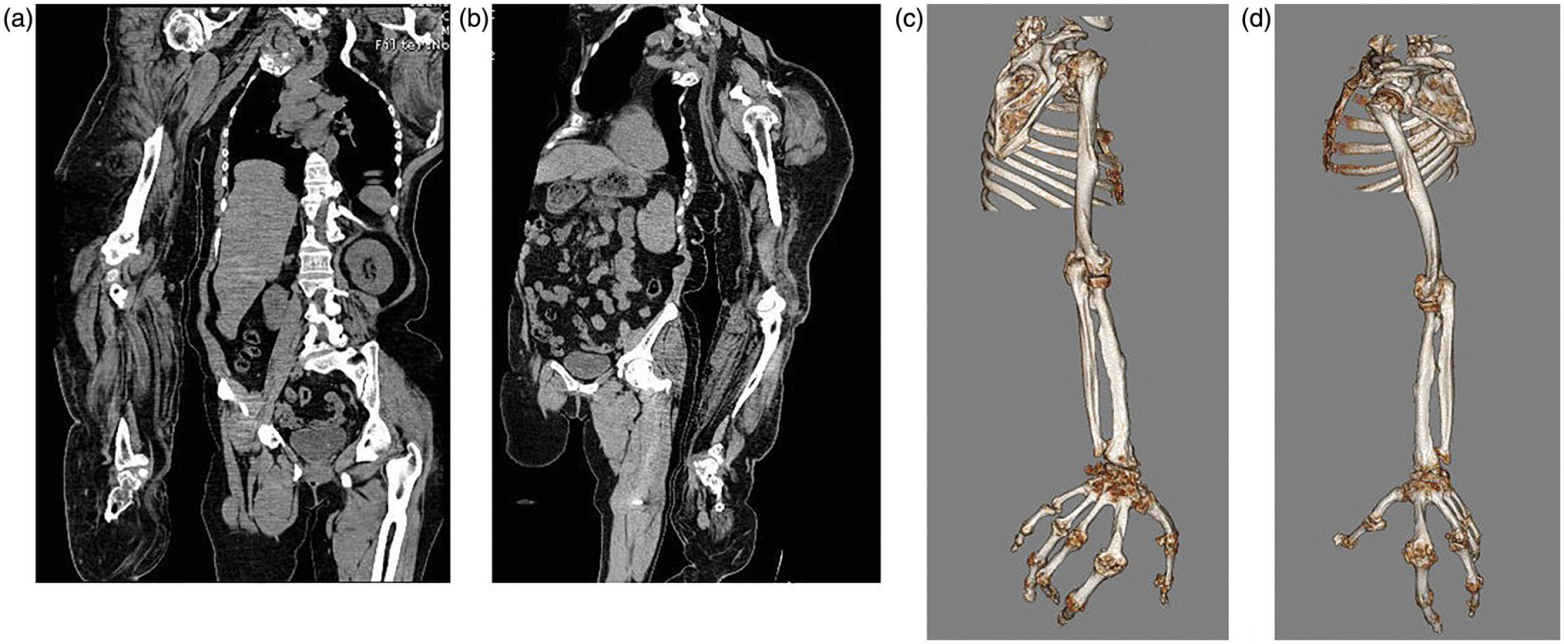
(a–d) CT scans: Adipose tissue hyperplasia was salient in the subcutis and intermuscular stroma of both upper extremities, with bilateral macromelia and macrodactyly. The ring and little fingers of the left hand were not enlarged. Muscles and adipose tissue of the thigh were not atrophied or enlarged. The bilateral overgrowth of muscles of the shoulders, the arms, and the proximal forearms was salient. Adipose infiltration was noted in the stroma of the deltoid muscle and brachial biceps. Muscles of the right forearm were atrophied, and atrophy and adipose degeneration were salient in all muscles of the right hand.

Adipose tissue hyperplasia was salient in the subcutis and intermuscular stroma of both upper extremities, with bilateral macromelia and macrodactyly (Figures 3 and 4). The ring and little fingers of the left hand were not enlarged. Thyroid atrophy, splenomegaly, adipose infiltration in the pancreas, and bilateral renal microlithiasis were found. Muscles and adipose tissue of the thigh were not atrophied or enlarged.

##### Adipose tissue and muscles

Adipose tissue showing areas that were equivalent in radiodensity to normal tissue was localizedly enlarged in both upper extremities. The bilateral overgrowth of muscles of the shoulders, the arms, and the proximal forearms was salient. Adipose infiltration was noted in the stroma of the deltoid muscle and brachial biceps. Muscles of the right forearm were atrophied, and atrophy and adipose degeneration were salient in all muscles of the right hand.

##### The median nerve

The median nerve was more noticeable than usual in the areas of both upper extremities between the shoulders and the wrist joints, and its interior showed a low-density area on the CT scans that was considered to be fat. The median nerve was enlarged in the carpal tunnel of both upper extremities.

##### Bones and joints

Deformation and ankylosis were bilaterally found in the shoulder, elbow, wrist, carpal, and finger joints. The ring and little fingers of the left hand showed no bone deformation. The lengths of major bones of the upper extremities measured by CT are shown in Table 1.

**Table 1.** Comparisons of the lengths of the humerus, radius, and ulna of our patient on the computed tomography scans with those described in a prior anthropological study.

	Fixed points of measurement	Right	Mean ± SD, right (Sasou and Hanihara [35])	Left	Mean ± SD, left (Sasou and Hanihara [35])
Humerus (base point at the acromion), mm	Acromion-lateral epicondyle	317	None	314.8	None
Humerus (base point at the bone head), mm	Superior border of the humeral head-lateral epicondyle	297.6	279.2 ± 14.82	297.6	276.2 ± 13.57
Radius, mm	Superior border of the radial head-styloid process	240.7	204.0 ± 12.13	233	203.9 ± 11.54
Ulna (no description), mm	Superior border of the ulnar head-styloid process	254.9	220.2 ± 12.40	255.9	219.7 ± 12.44

The lengths of the humerus, radius, and ulna of our patient were longer than those reported by Sasou and Hanihara [35].

##### MRI

**Figures 5.**
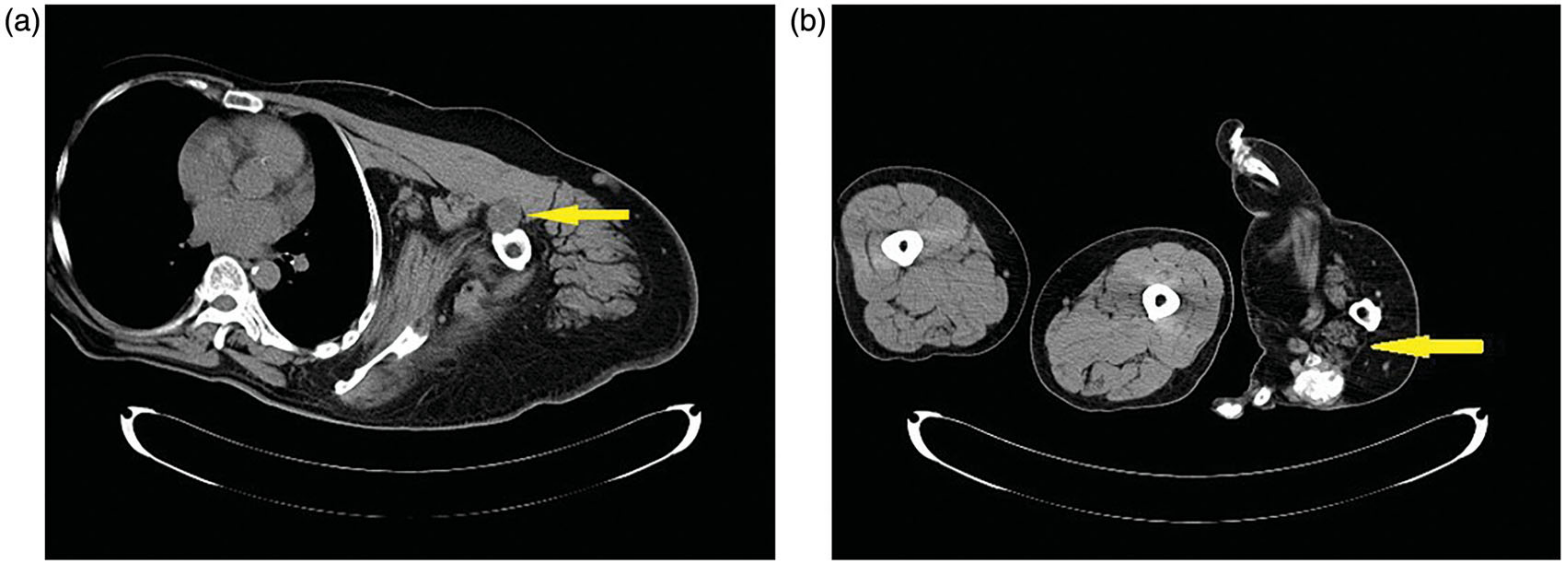
(a,b) MRI: MRI showed increased T2 signal in the interior of the median nerve, suggesting the presence of fat. The median nerve was enlarged of both upper extremities. Especially, adipose hyperplasia was salient in the carpal tunnel.

MRI provided findings similar to those obtained by CT (Figure 5). MRI showed increased T2 signal in the interior of the median nerve, suggesting the presence of fat. Adipose hyperplasia was salient in the carpal tunnel.

#### Diagnosis

Physical and imaging findings indicated bilateral and disproportional overgrowth that was localized in the upper extremities. Diagnostic imaging afforded findings suggestive of adipose infiltration in the median nerve. The patient had no family history. Together, the patient was diagnosed with MDL.

## Discussion

MDL is a rare, nonhereditary, and congenital disorder showing the disproportional, progressive overgrowth of mesenchymal tissues including adipose tissue, soft tissue, and bone. In 1925, a patient with MDL of the lower extremities was first reported by Feriz [1]. In 1967, Barsky classified MDL into two types: static type showing overgrowth in proportion to the patient’s growth; and progressive type showing disproportional, progressive overgrowth not in proportion to the patient’s growth [5]; the latter is considered rarer to develop [6]. Although these two types differ in progression, none of case reports has discussed whether they are of the same pathological entity. Various hypotheses on the etiology of MDL have been postulated, including lipomatous degeneration, fetal circulatory abnormality, damage of the limb bud, somatic cell changes in intrauterine life, and hypertrophy of the involved nerves [7–9]. A recent reports showed an association of MDL with mutations in the PIK3CA gene [10]. PIK3CA-associated segmental overgrowth is confirmed typically in affected tissues. Though, failure to detect a PIK3CA pathogenic variant does not exclude a clinical diagnosis of the PIK3CA-associated segmental overgrowth disorders in individuals with suggestive features [11]. Consequently, it is very difficult to separate these entities. Namely, the etiology of MDL remains unknown.

### Diagnosis

Usually, MDL is diagnosed based on family history, physical findings, and results from diagnostic imaging [12]. The relatives of the patient with MDL show no congenital anomalies of the extremities because the disorder is nonhereditary in nature. Physical examination indicates the disproportional overgrowth of the extremities in both longitudinal axis and circumference. Diagnostic imaging includes radiography, ultrasonography, CT, and MRI [13]. Among imaging modalities, MRI has been well recognized for its usefulness in a number of case reports [14,15]. Imaging findings include the localized overgrowth of soft tissues (e.g. adipose tissue that is equivalent in signal intensity to normal tissue) and bone tissues, adipose infiltration in the nerves, and ankylosis [6,13]. In a patient who undergoes invasive treatment, furthermore, MDL may be diagnosed along with histopathological examination that demonstrates the presence of fiber-scattered adipose tissue in soft tissues including the nerves, bone marrow, and other tissues [2,12,13]. Furthermore, neurophysiological examination may be conducted additionally that measures nerve conduction velocity and detects reductions in motor and sensory conduction velocities, blocked or reduced segmental conduction, and other changes [6].

The differential diagnosis of MDL includes fibrolipomatous hamartoma, lymphangiomatosis, hemangiomatosis, Klippel-Trenaunay-Weber syndrome, and Proteus syndrome [16–20]. Among them, the concurrence of MDL and fibrolipomatous hamartoma in the upper extremities has frequently been reported [21–23]; however, the association thereof remains unclear [6]. Other concurrent disorders include clinodactyly, syndactyly, polydactyly, and symphalangism [4,24]. The some authors consider that MDL as a localized form of Proteus syndrome in reports before 2000 [25–27]. Furthermore, there are some confusion of macrodystrophia lipomatosa and fibrolipoma of nerve [9,28]. Although, the most authors believe that the gross changes have as histopathological substrate the proliferation of mesenchymal elements with an excess of adipose/fibro-adipose tissue in the dermis in MDL [28].

### Treatment

Dysfunction, pain, and cosmetic issues caused by MDL are critical for the patient. However, no radical treatment is available at present [2,12,17]. Some case reports described the amputation of the affected site and debulking surgery [9]. However, no definite therapeutic outcomes have been obtained. Some patients showed improvements in cosmetics and function, and others did not. Several authors mention that overgrowth disorders could be treated in younger patients with the reconstructive surgical intervention and amputation [17,29–31]. On the other hand, according to another reports, recurrence rate of surgery is 33–60% [32]. Probably, several successive procedures will be necessary in order to achieve the desired outcome at the completion of skeletal growth [29–31]. The amount and extension of the interventions will be tailored depending on the evolution of the deformity and the degree of functional impairment [29]. In 2015, a study described the algorithm for the diagnosis and treatment of MDL [12]. According to the algorithm, follow-up of the patient is recommended without performing surgery when finding two or more of the following conditions: onset prior to puberty, affecting 1 or more fingers or toes, and medical emergency [12].

### Presentation of our patient and literature review

We found 131 cases of MDL in 102 case reports and articles according to PubMed that covered published articles between 1952 and 2019. In the vast majority of cases, MDL was unilateral and developed in the fingers or toes. Bilateral MDL developed localized in the fingers or toes of two patients [2,33]. On the other hand, MDL unilaterally affecting the entire upper extremity developed in four paitents [12,34]. To the best of our knowledge, we are the first to report on a case of MDL bilaterally affecting the entire upper extremity. Although PubMed did not list any case of MDL that extended to the same areas of lesions as that of our patient, we diagnosed the patient with MDL based on history of present illness, family history, physical findings, and imaging findings.

Our patient had the macroscopically apparent, disproportional overgrowth of the upper extremities compared with the trunk. To clarify bone overgrowth that is not obvious macroscopically, we measured the lengths of the upper extremity bones on the CT scans. Since we could not make comparisons between the affected upper extremity and the unaffected counterpart because our patient had bilateral MDL, we compared bone lengths measured in our patient with the standard lengths of bones (i.e. lengths of bones calculated from height) of Japanese women in an anthropological article [35] (Table 1). We employed the anthropological data of Japanese women because we could not find any anthropometric data of the race to which our patients belong. This is the first attempt to compare bone lengths measured on the CT scans with calculated standard bone lengths. The increased lengths of the humerus, radius, and ulna were apparent in our patient. However, we had the impression that long bones did not so elongate as estimated from overgrowth in appearance.

At present, a disorder showing the localized overgrowth of mesenchymal tissues in the extremities is diagnosed as MDL. However, MDL reported to date is diverse: static MDL that does not progress when the patient’s growth ceases; progressive MDL that advances regardless of the patient’s growth; MDL that is localized in the fingers or toes; and MDL that extends to the entire upper or lower extremities. We consider that whether these variants of MDL are of the same pathological entity is debatable. Furthermore, we found a case report that described MDL which skipped to the medial nerve [6]. Therefore, we could not solve the issue about whether or not MDL localized in the fingers or toes is simply detected in the course of its progression to a disorder that extends to the entire upper or lower extremities. We consider that our patient, who showed the overgrowth of mesenchymal tissues in areas not reported to date, potentially attributes to the elucidation of MDL pathology. Comprehensive gene expression analysis by next-generation sequencing on MDL in the future will allow gene expression profiling and will enable a novel therapeutic strategy including gene therapy.

## Limitations

Our patient did not desire any invasive tests, which impeded us from making auxiliary diagnoses (i.e. histopathological diagnosis and immunohistochemical diagnosis).

## Conclusions

We encountered a patient with MDL that extended bilaterally to the entire upper extremity – a case of MDL that has never been reported in a medical journal, to the best of our knowledge. We reported on the case, along with literature review.

Note: The patient provided consent to be reported in a case report.
